# Mechanisms and safety evaluation of stir-fried atractylodis macrocephalae rhizoma with aurantii fructus in the treatment of inflammatory bowel disease: a study based on organ-on-a-chip model

**DOI:** 10.3389/fphar.2025.1708719

**Published:** 2026-01-29

**Authors:** Weidong Zhang, Chanming Liu, Wei Chen, Yueqin Zhu, Zhengrong Gu, Ping Xue, Xin Zhuang, Yuan Wei, Jiexian Ye, Xi Xu, Jing Zhang, Zaozao Chen, Zhongze Gu, Feng Hua

**Affiliations:** 1 Changzhou Hospital of Traditional Chinese Medicine, Changzhou, China; 2 Changzhou City Food, Drug, and Fiber Quality Supervision and Inspection Center, Changzhou, China; 3 Jiangsu Avatarget Biotechnology Co., Ltd., Suzhou, China; 4 State Key Laboratory of Bioelectronics, School of Biological Science and Medical Engineering, Southeast University, Nanjing, China

**Keywords:** atractylenolide I, IL-17C, inflammatory bowel disease, organ-on-a-chip, stir-fried atractylodis macrocephalae rhizoma with aurantii fructus

## Abstract

**Background:**

Stir-fried atractylodis macrocephalae rhizoma (AMR) with aurantii fructus (AF) (SFALCA), a classical prescription of traditional Chinese medicine (TCM), has been widely used for promoting gastrointestinal health for centuries, with multiple pharmacological properties such as anti-tumor, anti-inflammatory, anti-aging, antioxidant, and antibacterial effects. Inflammatory bowel disease (IBD) is an immune-mediated chronic gastrointestinal inflammatory disorder that causes long-term distress to patients. Despite its widespread use, the specific effects of SFALCA on intestinal barrier function and underlying mechanisms remain unclear. Furthermore, interspecies discrepancies in animal studies and the physiological constraints of existing *in vitro* models synergistically limit the translational potential of current findings on this botanical combination.

**Methods:**

To investigate the active components, mechanisms of SFALCA in treating IBD and assesses its safety. We designed an innovative organ-on-a-chip system to simulate the human intestinal and liver environment. By stimulating with LPS/PMA, we established an *in vitro* IBD model and intervened with SFALCA and its extracts. Immunofluorescence staining was used to evaluate the success of the model. TEER measurements were employed to assess the integrity of tight junctions. Alcian Blue staining characterizes the expression of acidic mucins in HT-29 cells. The levels of inflammatory cytokines and the human albumin were measured using ELISA kits. The cytotoxicity of TCM to liver was evaluated by CellTiter-Glo® 3D test according to the manufacturer’s instructions. Flow cytometric analysis was used to detect the polarization of macrophages in intestinal inflammation model after drug treatment. RNA-seq analysis was used to identify key targets and pathways.

**Results:**

The results showed that SFALCA and its extracts significantly increased transepithelial electrical resistance (TEER) and Zonula occludens-1 (ZO-1) expression, while inhibiting LPS/PMA-induced IL-6 levels and the proportion of M1 macrophages. Further analysis revealed that the main active components of SFALCA, Atractylenolide I and Naringin, exert anti-inflammatory effects by inhibiting the Interleukin-17C (IL-17C) mediated positive feedback loop. Additionally, organ-on-a-chip technology confirmed that SFALCA showed no significant toxicity to the liver.

**Conclusion:**

In conclusion, this study elucidates the active components and mechanisms of SFALCA in treating IBD and assesses its safety, providing a reliable *in vitro* platform for future therapeutic strategies.

## 1 Introduction

Stir-fried atractylodis macrocephalae rhizoma (AMR) with aurantii fructus (AF) (SFALCA), a classical prescription of traditional Chinese medicine (TCM), has been widely used for promoting gastrointestinal health for centuries, with multiple pharmacological properties such as anti-tumor, anti-inflammatory, anti-aging, antioxidant, and antibacterial effects (Wang et al., 2009; Wang et al., 2014; Li et al., 2014; Li et al., 2012; Shu et al., 2017). These herbs are commonly used in single or combined formulations to treat gastrointestinal disorders such as diarrhea and bloating. AMR has significant anti-gastritis effects *in vivo* and *in vitro*, which can be associated with the inhibition of the Akt/IκBα/NF-κB signalling pathway (Hossen et al., 2021). AF can take effects on inflammatory bowel disease (IBD) through anti-inflammation and inhibition of intestine muscle contraction (He et al., 2018). Their main active ingredients include flavonoids, alkaloids, atractylenolide I–III, and polysaccharides. As one of the unique formulations of the Menghe Medical School, SFALCA has shown potential in the treatment of IBD. However, TCM with its complex composition and diverse active substances, along with specificities in herbs, origins, preparation, and extraction, often encounters obstacles to its modernization and internationalization, especially concerning potential hepatotoxicity (Rao et al., 2021; Dong et al., 2020; Li et al., 2022). Therefore, evaluating the hepatotoxicity of SFALCA and exploring its effects and mechanisms in treating gastrointestinal diseases not only clarifies its safety but also provides scientific evidence for its clinical application. This is of great importance for ensuring the safety of clinical use and advancing the modernization and internationalization of TCM.

Inflammatory bowel disease (IBD) is a chronic gastrointestinal condition characterized by widespread inflammatory damage to the intestinal epithelium. Typical diseases include ulcerative colitis (UC) and Crohn’s disease (CD), with millions of patients worldwide (Piovani et al., 2020). Although other types, such as microscopic colitis and indeterminate IBD, have lower incidence rates, their severity should not be underestimated (Derkacz et al., 2018; Burke et al., 2021). The pathogenesis of IBD is highly complex, involving genetic susceptibility and interactions with “Western lifestyle” factors such as high-fat diets, overuse of nonsteroidal anti-inflammatory drugs, and antibiotic use (Kofla-Dłubacz et al., 2022; Faye et al., 2023; Rizzello et al., 2019). These factors cause cumulative micro-damage to the intestinal epithelial barrier, leading to dysbiosis and activation of local immune cells, particularly macrophages, which release pro-inflammatory mediators (such as TNF-α, IL-6, IL-1β), ultimately triggering uncontrolled inflammation (Neurath, 2019). Typical symptoms of IBD include diarrhea, abdominal pain, rectal bleeding, fatigue, and weight loss. Studies suggest that an imbalance in macrophage polarization (M1/M2 phenotype) plays an important role in IBD development. M1 macrophages (polarized by IFN-γ/LPS) drive pro-inflammatory responses, while M2 macrophages (polarized by IL-4/IL-13) exert immune-regulatory functions (Boutilier and Elsawa, 2021).

Current IBD research models include the common chemically-induced colitis mouse models, such as those using dextran sulfate sodium (DSS) (Katsandegwaza et al., 2022; Li et al., 2024), and early-stage *in vitro* models composed of single-cell types. While these models are useful for efficacy evaluation, interspecies differences and the complexity of cellular mechanisms in animal models often fail to accurately simulate the full range of IBD manifestations in humans. Furthermore, simple single-cell *in vitro* models lack effective monitoring of immune cell activation and are unable to simulate the complex pathological processes of IBD (Cobrin and Abreu, 2005). In contrast, the Transwell® co-culture model based on human colon cancer cells (Caco-2) and monocytic leukemia cells (THP-1) can simulate macrophage-mediated inflammatory responses (Cobrin and Abreu, 2005), but it still cannot fully replicate physiological functions such as nutrient exchange, oxygen delivery, and waste clearance in the intestine. Recently, the human intestinal-immune organ-on-a-chip model, using microfluidics to simulate nutrient and waste exchange, has provided a more realistic intestinal disease model (Aziz et al., 2017; Marrero et al., 2021; Pimenta et al., 2022; Verhulsel et al., 2021).

In this study, we developed a human intestinal chip model by co-culturing human intestinal epithelial cells (Caco-2), human intestinal goblet cells (HT29-MTX), human umbilical vein endothelial cells (HUVEC), and human acute monocytic leukemia cells (THP-1) derived macrophages. We induced the IBD model using LPS and PMA. The model addresses the limitations of conventional intestinal models, such as the lack of diverse cell types, immune components, and non-physiological factors, thereby establishing a more physiologically relevant *in vitro* model. The primary objective of the study was to evaluate the effects of SFALCA in IBD treatment and its active ingredients, and to identify key targets and mechanisms through RNA-seq analysis. Additionally, we established a liver microenvironment chip containing human hepatocellular carcinomas cells (HepG2), liver sinusoidal endothelial cells, and hepatic stellate cells to assess the liver safety of SFALCA. This study not only provides a new technological platform for toxicity assessment and pharmacological mechanism research of TCM, but also offers important theoretical and practical references for promoting the application of organ-on-a-chip technology in TCM.

## 2 Materials and methods

### 2.1 Cell culture

Caco-2 were grown in Dulbecco’s Modified Eagle Medium (DMEM, Gibco, United States, 11965-092) medium containing 20% fetal bovine serum (FBS, Gibco, United States, A5669701) and 1% penicillin-streptomycin antibiotics (P/S, Gibco, United States, 15140-122). HT-29 were grown in DMEM medium containing 10% FBS and 1% P/S. LX-2 and HepG2 cells were grown in DMEM medium containing 10% FBS and 1% P/S. Human THP-1 monocytic cells were cultured in T-25 flasks (Corning, United States, 430639) in RPMI 1640 (Gibco, United States, 11875-093) containing 10% FBS, 1% P/S, and 50 µM β-mercaptoethanol (Absin, China, ABS9592). HUVEC and Human Liver Sinusoidal Endothelial Cells (LSEC) were cultured in Endothelial Cell Medium (ECM, Sciencell, United States, 1001).

All cells were maintained in a 5% CO_2_ atmosphere at 37 °C and both medium were changed every 2–3 days. The cells were passaged by trypsin-EDTA (Gibco, United States, 15050065) at a ratio of 1:3 when the confluence reached 80%.

### 2.2 TCM and active components

Atractylodis Macrocephalae Rhizoma *(Atractylodes macrocephala* Koidz.) (AMR) (batch: 231202, Origin: Anhui) and Aurantii Fructus (*Citrus aurantium* L.) (AF) (batch: 231055, Origin: Jiangxi) were purchased from Suzhou Tianling Chinese Traditional Medicine Slice Co. Ltd. Atractylenolide I (batch: HY-N0201, concentration: 99.82%) and Naringin (batch: HY-N0153, concentration: 93.5%) were purchased from Medchemexpress Co. Ltd.

### 2.3 Sample preparation

Fifteen grams of AF were accurately weighed and decocted with distilled water twice. The resulting decoctions were combined and concentrated to a final concentration of 0.3 g/mL, yielding the AF aqueous extract. Subsequently, the prepared AF aqueous extract was thoroughly mixed with 100 g of AMR, and the mixture was moistened at room temperature for 25 minutes, followed by stir-frying at 130–140°C for 15 minutes to prepare SFALCA. For subsequent experimental use, SFALCA was decocted with distilled water three times; the decoctions were combined, and the pooled mixture was freeze-dried to obtain SFALCA freeze-dried powder.

### 2.4 THP-1 differentiation into macrophages

THP-1 cells were differentiated into resting macrophages according to the protocol described by Li et al. (2017). Briefly, THP-1 cells were resuspended in a culture medium containing 100 ng/mL PMA (Sigma, Japan, P1585) for 48 h, and then the differentiated THP-1 cells were used for subsequent studies.

### 2.5 Construction of intestinal-immune chip

As shown in Figure 2A, HUVEC cells were first seeded onto the lower membrane of the chip. On the next day, Caco-2 cells and HT-29 cells were co-cultured in the upper channel of the chip. After 3–4 days of culture, when the TEER value of the intestinal part reached 400 Ω·cm^2^, M0-type immune macrophages were added to the lower channel of the chip. Following 24 h of co-culture with M0 macrophages, the gut-immune chip model was established and could be used for subsequent experiments and analysis. After each cell seeding and adhesion, the chip was placed in a dynamic rocking perfusion device set to continuous mode, with a rocking angle of 7° and a rocking time of 8 min (Rajasekar et al., 2020).

### 2.6 Construction and treatment of intestinal-immune inflammatory model

After constructing the intestinal-immune chip model, a medium containing LPS and PMA mixture was added to the upper channel to induce an intestinal inflammatory model for 8 h. Upon successful construction of the inflammatory model, drug-containing medium was added to both upper and lower channels for 48 h of culture. Blank control group: 0.1% DMSO (Sigma, United States, D265); Positive drug group: α-lipoic acid (ALA, MCE, United States, 1O77-28-Z) 100 μmol/mL; Raw AL group: 100 μg/mL; SFALCA group: 100 μg/mL; Atractylenolide I group: 100 μmol/mL; Naringin group: 100 μmol/mL; Combined drug group: Atractylenolide I 100 μmol/mL and Naringin 100 μmol/mL.

### 2.7 Construction of liver chip model

**FIGURE 1:**
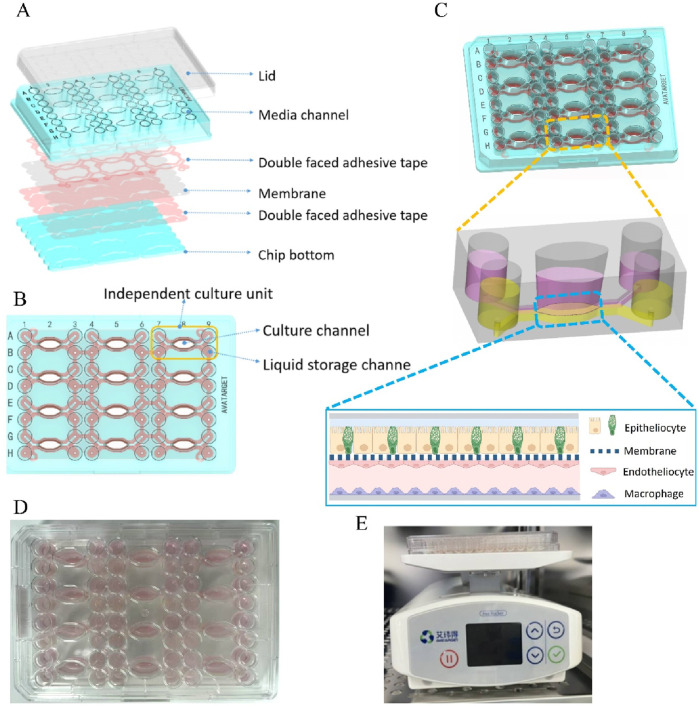
Design and assembly of intestine on a Chip. **(A)** Exploded view of the chip. **(B)** Vertical view of the chip, including 12 independent culture units. **(C)** Axonometric drawing of the chip. **(D)** Appearance of the chip. **(E)** Dynamic culturing of the intestine-on-a-chip in the incubator.

As shown in Figure 1C, LSEC cells were first seeded into the chip. On the next day, HepG2 cells and LX-2 cells were co-cultured in the upper channel of the chip. The liver chip model was completed after cell fusion.

### 2.8 Immunofluorescence analysis

The chips were firstly washed by flowing PBS (Solabio, China, P1020) through the upper and lower channels, and then fixed with 4% paraformaldehyde (PFA, Biosharp, BL539A) for 30 min. The chips were permeabilized with 0.3% Trition X-100(Beyotime, China, P0096) and followed by blocked with blocking solution (Beyotime, China, P0126). Subsequently, HepG2 cells were incubated with goat anti-Albumin (Bethyl, China, A80-229A, 1:500 diluted), LSEC and HUVEC cells were incubated with rabbit anti-CD31 (Abcam, United Kingdom, ab32457, 1:200 diluted), LX-2 cells were incubated with mouse anti-α-SMA (Abcam, United Kingdom, ab7817, 1:200 diluted), and Caco-2 cells were incubated with rabbit anti-ZO-1 (Invitrogen, United States, 33-9188, 1:200 diluted), respectively, at 4 °C overnight. After washing with PBS, samples were incubated with secondary antibodies 488-conjugated Donkey Anti-Goat IgG (Jackson, United States, 705-545-003, 1:1000 diluted), 488-conjugated Goat Anti-Rabbit IgG (Jackson, United States, 705-545-152, 1:1000 diluted), 594-conjugated Donkey Anti-Mouse IgG (Jackson, United States, 715-585-150, 1:1000 diluted), and 594-conjugated Donkey Anti-Rabbit IgG (Jackson, United States, 711-585-152, 1:1000 diluted) at room temperature. Fluorescent images were acquired by the microscope (IX-83, Olympus, Japan).

### 2.9 Alcian blue staining

Alcian Blue (Beyotime, China, C0155M) staining characterizes the expression of acidic mucins in HT-29 cells. After constructing the intestinal chip model, wash the model with ddH_2_O, then fix it with 4% PFA at room temperature for 30 min. After fixation, wash the model three times with ddH_2_O, 5 min each. Next, add 200 μL of Alcian blue staining solution to the top layer and stain at room temperature for 1 h. Following the staining, wash the model three times with ddH_2_O to remove the staining solution, 5 min each. Then, add 200 μL of nuclear fast red staining solution and stain at room temperature for 5 min. Afterward, wash the model three times with ddH_2_O to remove the staining solution, 5 min each. Finally, observe and photograph the model under a microscope.

### 2.10 ELISA assays

The supernatant of the cell culture medium mixture in the upper and lower layers of the model was collected by centrifugation at 3,000 × g for 15 min. The levels of IL-6(Proteintech, United States, KE00139), IL-10 (Proteintech, United States, KE00170), and the human albumin (Proteintech, United States, KE00076) in the supernatant was determined using corresponding ELISA kits and followed the manufacturer’s instructions. Eventually, absorbance at 450 nm was recorded by a microplate reader (Thermo Fisher Scientific, United States).

### 2.11 Cell viability assay

The cytotoxicity of TCM to liver was evaluated by CellTiter-Glo® 3D (promega, United States, G9683) test according to the manufacturer’s instructions. The upper and lower layers were added with medium containing drugs for 48 h after the construction of liver chip model. The concentration of raw AL and SFALCA was 100 μg/mL; the concentrations of ALA, atractylenolide I, naringin, and the combination medication group (atractylenolide I + naringin) were all 100 µM. After treatment, CellTiter-Glo® 3D with the same volume as the medium was added to the upper and lower layers of the model respectively. The contents were vigorously mixed on a plate shaker for 5 min, incubated at room temperature for 25 min, transferred to opaque-walled plates, and the luminescence recorded using a multifunctional microplate reader (Thermo Fisher Scientific, United States).

### 2.12 Transendothelial electrical resistance (TEER) measurement

During the construction of the intestinal model, TEER measurements are employed to assess the integrity of tight junctions. A TEER value of 400 Ω·cm^2^ or higher indicates adequate barrier function in the model. In the inflammatory intestinal model, TEER values are measured at 2, 4, 6, and 8 h following the addition of the stimulant. A significant decrease in TEER is considered a key indicator of successful model establishment. To evaluate the repair effects of a test substance on the intestinal model, TEER measurements are taken both before and 48 h after the addition of the test substance.

Before each TEER measurement, the quick electrode is cleaned by immersing it in 75% alcohol for 30 s, followed by a 30-s rinse in sterile ddH_2_O. The electrode is then inserted into the culture medium within the chip well, ensuring that the short electrode contacts the apical chamber and the long electrode contacts the basolateral chamber. Care must be taken to avoid direct contact between the electrode and the cell layer to prevent disrupting the barrier. A well containing only medium and no cells is measured as a blank control each time. The effective growth area of the intestinal chip is 0.33 cm^2^, and the measured TEER values are calculated using the following formula before statistical analysis (Nazari et al., 2023).

$$\text{TEER}=\left(R_{\text{Sample}}-R_{\text{Control}}\right)\times \text{Membraner Area}$$

### 2.13 Flow cytometry analysis

Flow cytometric analysis was used to detect the polarization of macrophages in intestinal inflammation model after drug treatment. The differentiated THP-1 cells were detached with trypsin-EDTA and collected by centrifugation at 1,000 rpm for 5 min. For cell surface antigen staining, macrophages were stained with PE anti-Human CD80 (4A Biotech, China, FHN80-01-100) at 4 °C for 30 min. After washing, the cell fixation solution was added and the samples were incubated at room temperature in the dark for 10 min. Subsequently, a membrane-permeabilizing agent was added to permeabilize the cell membranes for 15 min. For intracellular antigen staining, macrophages were stained with PE/Cy7 anti-Human CD206 (4A Biotech, China, FHN206-01-100) at room temperature for 30 min. The labeled cells were analyzed by flow cytometry with a FACScalibur (Beckman, United States), and the data were processed with FlowJo software.

### 2.14 RNA extraction and RNA-sequencing analysis

Total RNA was isolated from samples using the RNAiso Plus Kit (Takara, Beijing, China) following the manufacturer’s protocol. RNA concentration and integrity were assessed using a Qubit 4.0 Fluorometer (Thermo Fisher Scientific, United States) and an Agilent 2100 Bioanalyzer (Agilent Technologies, United States), respectively. RNA-seq libraries were constructed with the VAHTS Stranded mRNA-seq Library Prep Kit for Illumina (Vazyme Biotech, China). Sequencing was performed on an Illumina NovaSeq 6000 platform (Illumina, United States) with 150-bp paired-end reads. Raw sequencing reads were subjected to quality control using Fastp (version 0.23.2) to remove low-quality sequences and adapter contamination. High-quality reads were aligned to the GRCh38 human reference genome via HISAT2 (version 2.2.1). Transcript assembly and quantification were conducted using StringTie (version 2.2.1), followed by normalization of expression data with the DEGseq R package (version 1.48.0) for differential expression analysis.

### 2.15 Statistical analysis

Ordinary two-way ANOVA and unpaired *t*-test were used to examine differences between quantitative values. Statistical significance is indicated by a *p* value of less than 0.05, where *p < 0.05, **p < 0.01, ***p < 0.001, ****p < 0.0001. Data are presented as mean ± SD. GraphPad Prism software version 8 (GraphPad Software, San Diego, CA, United States) was used for all statistical analyses.

## 3 Results

### 3.1 Design and assembly of intestine on a chip

The design of the intestine-on-a-chip is shown in Figure 1. Briefly, polycarbonate (PC) was processed by a computer numerical control (CNC) machine to obtain various parts of the chip and then cleaned with ethanol and double distilled water. After drying, the various parts of the chip and polyethylene terephthalate (PET) membrane with a pore size of 0.45 μm were assembled into a whole using pressure-sensitive double-faced adhesive tape (Figure 1A). The overall dimensions of the chip are 127.80 × 85.50 × 17.2 mm, with a membrane cultivation area of 0.33 cm^2^. The chip contained 12 independent culture units for the high-throughput experiment (Figures 1B–D). Each culture unit was composed of liquid storage channels and culture channels (Figure 1B). After high-temperature sterilization and drying of the chip, epithelial cells were inoculated on the upper layer and endothelial cells and macrophages were inoculated on the lower layer (Figure 1C). The intestine-on-a-chip was placed in an incubator (37 °C and 5% CO_2_) on a rocker platform (AVATARGET, China) with a seven inclination and 8 min cycle time to allow bidirectional flow as previously described (Figure 1E) (Rajasekar et al., 2020).

### 3.2 Construction and characterization of intestinal model

**FIGURE 2:**
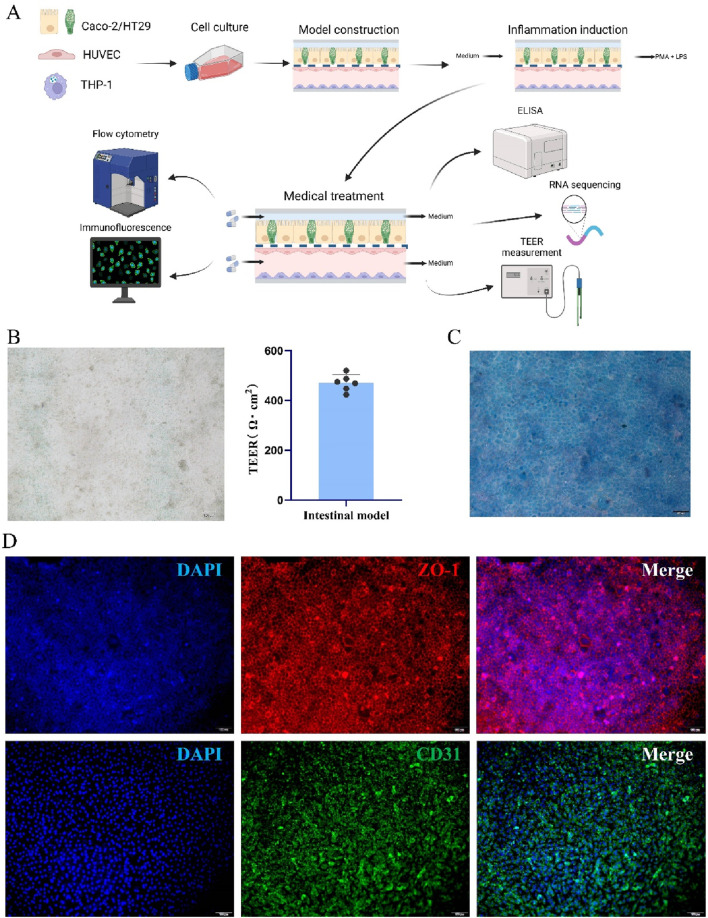
Construction and characterization of intestinal model. **(A)** Construction and assessment of the intestinal-immune model. **(B)** Bright-field images and TEER values of the model. **(C)** Alcian blue staining of the model. **(D)** Immunofluorescence staining of ZO-1 and CD31 in the model.

The intestinal model was constructed using four cell types: intestinal epithelial cells Caco-2 and HT-29, endothelial cells HUVEC, and M0 macrophages derived from THP1 cells. After inflammation modeling and drug treatment, several assays, including TEER measurement, immunofluorescence staining, ELISA, flow cytometry, and RNA sequencing, were performed to evaluate the effects of the drug on intestinal barrier repair (Figure 2A).

To begin with, we conducted a thorough evaluation of the constructed intestinal model by employing various techniques to assess both the growth status of the cells and the expression of key proteins within the model. As shown in Figure 2B, after cultivation, the cells in the model were evenly distributed and exhibited good growth, with an average TEER value exceeding 400 Ω·cm^2^, indicating robust barrier function. The Alcian Blue staining results (Figure 2C) revealed that HT-29 cells in the model grew and differentiated well and were capable of secreting mucus. The typical mucus secretion pattern observed in the staining indicated that the intestinal epithelial function was maintained. The immunofluorescence staining results further confirmed the successful construction of the model (Li et al., 2021) (Figure 2D). In Caco-2 cells, the expression of ZO-1 protein (a tight junction marker for intestinal epithelial cells) was significantly enhanced, with clear membrane labeling, indicating normal tight junction function in the epithelial cells (Wu et al., 2019). Similarly, high expression of CD31 (an endothelial cell marker) in HUVEC cells confirmed that the endothelial cells were functional within the model. The immunofluorescence results clearly demonstrated the interactions between Caco-2 and HUVEC cells and their stable biological functions in the intestinal model. Based on the above staining and assay results, it can be concluded that the cells in the model are evenly distributed, exhibit good growth, and express the relevant proteins normally (Baluk and McDonald, 2008). This indicates that the intestinal model was successfully constructed, providing a reliable experimental platform for subsequent drug screening and intestinal barrier repair research.

### 3.3 Establishment of intestinal inflammation model

**FIGURE 3:**
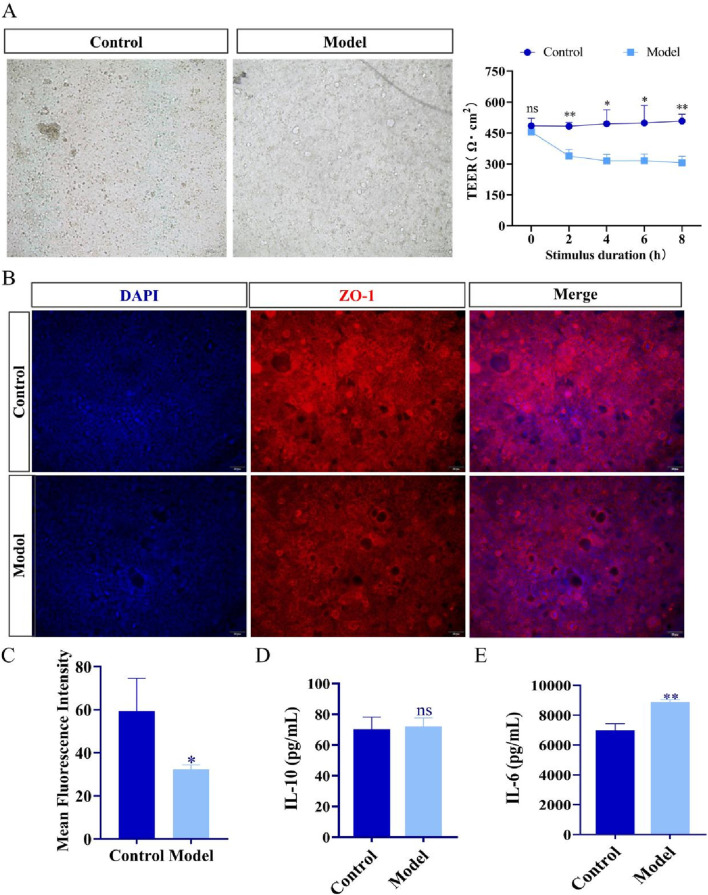
Establishment of intestinal inflammation model of TCM. **(A)** Representative bright-field images and TEER values of the intestinal model before and after stimulation with PMA and LPS (n = 3). Scale bar, 100 μm. **(B,C)** Representative immunofluorescence images of ZO-1 and quantitative analysis of the mean fluorescence intensity (n = 3). Scale bar, 50 μm. **(D)** ELISA kits were used to analyze the concentrations of IL-10 in each group (n = 3). **(E)** ELISA kits were used to analyze the concentrations of IL-6 in each group (n = 3)*p < 0.05; **p < 0.01; ns, not significant. Data are presented as mean ± SD.

The cells within the intestinal model exhibited dense growth, established tight junctions, and collectively formed an intact cellular barrier. After stimulation with PMA and LPS, the tight junctions between the cells were disrupted, compromising the integrity of the barrier, and the TEER value significantly decreased (Figure 3A). As depicted in Figures 3B,C, compared with the control group, the expression of ZO-1 was significantly reduced in the model group, further demonstrating the impairment of barrier integrity. The ELISA data indicated that, in the model group, the level of the pro-inflammatory cytokine IL-6 was significantly increased, while the level of the anti-inflammatory cytokine IL-10 did not show significant changes (Figures 3D,E). When taken together, these results demonstrate that the intestinal inflammation model was successfully established following induction with PMA and LPS.

### 3.4 Treatment of intestinal inflammation model with TCM

Initially, we evaluated the therapeutic effects of SFALCA at concentrations of 50 μg/mL, 100 μg/mL, and 200 μg/mL. Following treatment of the model, changes in the transepithelial electrical resistance (TEER) values were recorded and analyzed, and the expression of the tight junction protein ZO-1 was assessed via immunofluorescence staining (Wu et al., 2019). TEER serves as a key indicator for evaluating the integrity of intercellular tight junctions, where higher resistance values signify a stronger barrier function of the cell monolayer and is commonly used to monitor intestinal barrier status. ZO-1 is a pivotal tight junction protein highly expressed in the intestinal epithelium, directly associated with both the physical and functional integrity of the intestinal barrier (Wu et al., 2019).

According to Supplementary Figure S2, the most significant recovery of TEER values was observed in the model treated with 100 μg/mL and 200 μg/mL concentrations. Concurrently, these concentrations induced the highest fluorescence expression level of the barrier-associated protein ZO-1, indicating a restorative effect on the model’s barrier function post-treatment.

Furthermore, in the assessment of the pro-inflammatory cytokine IL-6 and the anti-inflammatory cytokine IL-10, the 100 μg/mL and 200 μg/mL concentrations also demonstrated the most substantial therapeutic efficacy, effectively suppressing inflammation in the model. Collectively, this evidence confirms the favorable therapeutic effects of both the 100 μg/mL and 200 μg/mL concentrations. Since the 200 μg/mL concentration did not exhibit a statistically significant superiority over the 100 μg/mL concentration across all evaluated metrics, the lower concentration (100 μg/mL) was selected for subsequent studies due to its comparable efficacy, potentially enhanced safety profile, and cost-effectiveness.

### 3.5 Treatment of intestinal inflammation model with TCM and its active components

Following the determination of the optimal therapeutic concentration of SFALCA, the extract’s bioactive components, including atractylenolide I and naringin, were incorporated into the experimental system for further evaluation.

**FIGURE 4:**
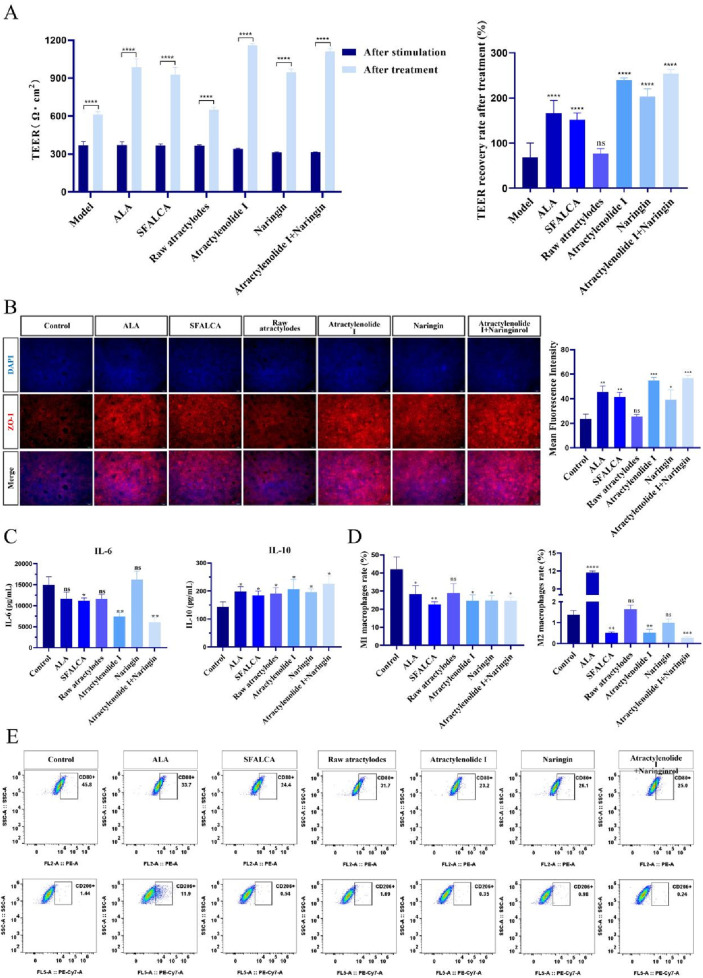
Treatment of intestinal inflammation model with TCM. **(A)** TEER values and their changes before and after treatment in the intestinal model (n = 3). **(B)** Immunofluorescence staining results of ZO-1 and statistical analysis after model treatment (n = 3). **(C)** Cytokine levels of IL-6 and IL-10 in the culture medium after treatment (n = 3). **(D)** Statistical analysis of the M1/M2 macrophage ratio after treatment (n = 3). **(E)** Flow cytometry results of the M1/M2 macrophage ratio after treatment. *p < 0.05; **p < 0.01; ***p < 0.001, ****p < 0.0001, ns, not significant. Data are presented as mean ± SD.

As shown in Figure 4A 48 h after the removal of the stimulus, TEER values significantly increased in all experimental groups, suggesting partial repair of the intestinal barrier in each group. Compared to the untreated control group, the raw atractylodes group did not show significant therapeutic effects. The recovery of TEER values in the other groups followed a descending order, from highest to lowest: the combination medication group: the atractylenolide I and naringin, the atractylenolide I group, the naringin group, the ALA group, and the SFALCA group.

After treatment, we fixed the models from each group and analyzed ZO-1 protein expression via immunofluorescence staining (Figure 4B). Statistical analysis of the average fluorescence intensity using ImageJ software revealed that the expression levels of ZO-1 closely mirrored the trends in TEER values. The raw AL group showed the lowest ZO-1 expression and TEER values among all the treatment groups, with no significant difference compared to the untreated group. The SFALCA group showed significant therapeutic effects, but these were less pronounced than those in the groups containing atractylenolide I. The combined treatment group showed the best overall performance, with minimal difference between the atractylenolide I and combined treatment groups, indicating that atractylenolide I plays a key role in intestinal barrier repair.

Additionally, we collected the culture media from each group to measure cytokine levels. The results in Figure 4C show that for the pro-inflammatory cytokine IL-6, the ALA, raw AL, and naringin groups did not differ significantly from the untreated group. However, the IL-6 levels in the SFALCA, atractylenolide I, and the atractylenolide I and naringin combined groups were significantly lower than those in the untreated group. Regarding the anti-inflammatory cytokine IL-10, all treatment groups showed significantly higher levels than the untreated group, with the combined treatment group exhibiting the highest IL-10 levels. Since cytokine levels are closely linked to macrophage polarization, we further analyzed the macrophages at the bottom of the model using flow cytometry to quantify the proportions of CD80 (M1 marker) and CD206 (M2 marker) macrophages (Figures 4D,E) (Liu et al., 2023; Li et al., 2024). As shown in the figures, only the raw AL group did not show a significant reduction in the proportion of M1 macrophages compared to the untreated group, while all other groups showed significant reductions in M1 macrophages, especially the SFALCA group. For M2 macrophage proportions, the ALA group exhibited a significantly higher proportion than all other groups, while the raw AL and naringin groups showed no significant difference from the control group. In contrast, the SFALCA, atractylenolide I, and combined treatment groups all exhibited significantly lower M2 proportions compared to the untreated group. The M2 macrophage proportions showed some variability, which will be further discussed in the following sections.

### 3.6 Establishment of liver models and safety evaluation of TCM and its active components

Based on the therapeutic effects observed for TCM and its extracts in intestinal inflammation models, it is necessary to further evaluate their potential hepatotoxicity to complete a comprehensive safety assessment. We have established a liver-on-a-chip model to systematically study the liver’s response at equivalent drug concentrations; this evaluation will provide an important basis for the safety of these medications.

**FIGURE 5:**
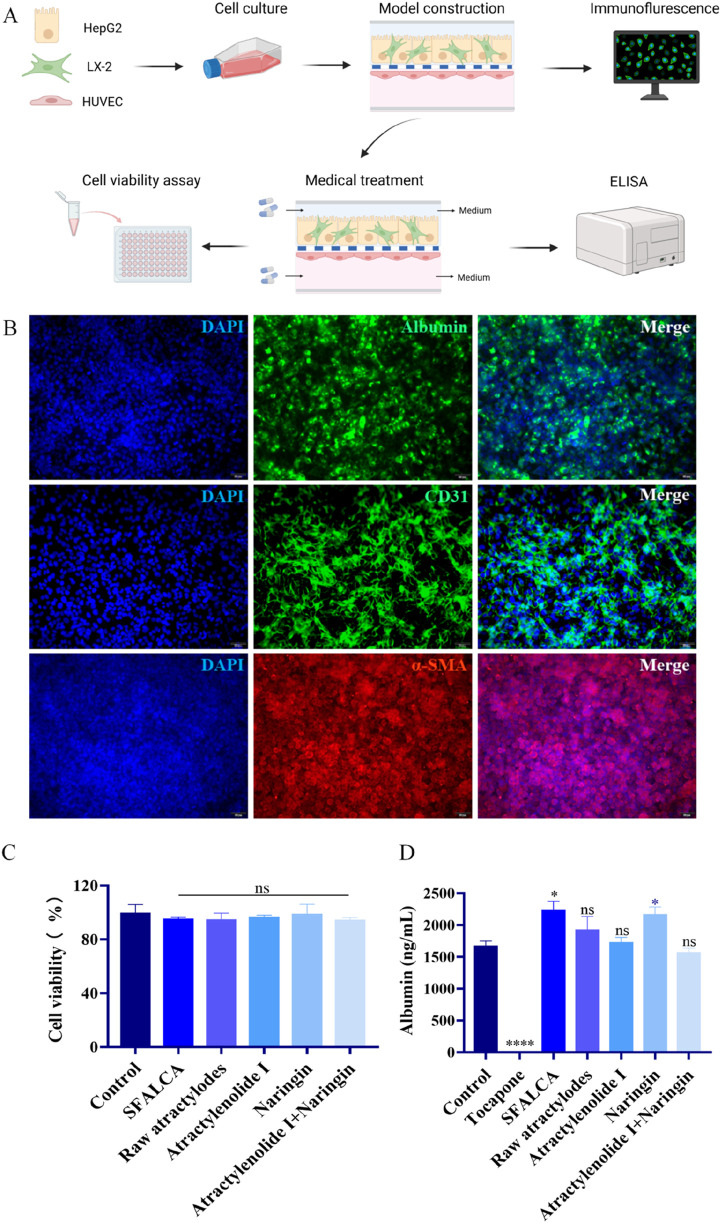
Establishment of liver model and safety evaluation of TCM. **(A)** Construction and assessment of the liver model. **(B)** Immunofluorescence staining was conducted to characterize the liver-on-a-chip model, with markers including albumin for HepG2 cells, α-SMA for LX-2 cells, and CD31 for LSECs. Scale bar, 50 μm. **(C)** The cytotoxic effects of TCM on liver model were assessed at 48 h post-treatment using the CellTiter-Glo® 3D assay (n = 3). **(D)** The expression levels of albumin in the liver model with TCM and the hepatotoxic drug tolcapone treatment (n = 3). *p < 0.05; **p < 0.01; ns, not significant. Data are presented as mean ± SD.

We constructed the liver model on the chip using HepG2 cells, LX-2 cells and LSEC cells (Chatterjee et al., 2018; Kwon et al., 2022), and characterized its relevant protein markers (Figure 5A). As shown in Figure 5B, HepG2 cells in the model highly expressed albumin (Nishida and Taniguchi, 2013), LX-2 cells highly expressed α-smooth muscle actin (α-SMA), and LSEC cells highly expressed CD31, indicating that the liver model had good cell growth and could normally express the relevant proteins (Nikolić et al., 2017; Mu et al., 2018; Poór et al., 2013). After treating the liver model with TCM, cell viability assays were performed. The results showed no significant differences between the TCM-treated groups and the control group (Figure 5C), indicating the absence of cytotoxicity. Additionally, ELISA results demonstrated that albumin levels (Kwon et al., 2022; Frojdenfal and Zuchowska, 2024; Ewart L et al., 2022) in the model sharply decreased following treatment with the hepatotoxic compound tocapone. In contrast, the combination of SFALCA and naringin significantly upregulated albumin expression, while no reduction in albumin levels was observed in other drug-treated groups (Figure 5D). These findings confirm that TCM formulations do not negatively affect hepatic albumin expression, thereby proving their safety.

### 3.7 Gene expression of intestinal inflammation model after treatment

**FIGURE 6:**
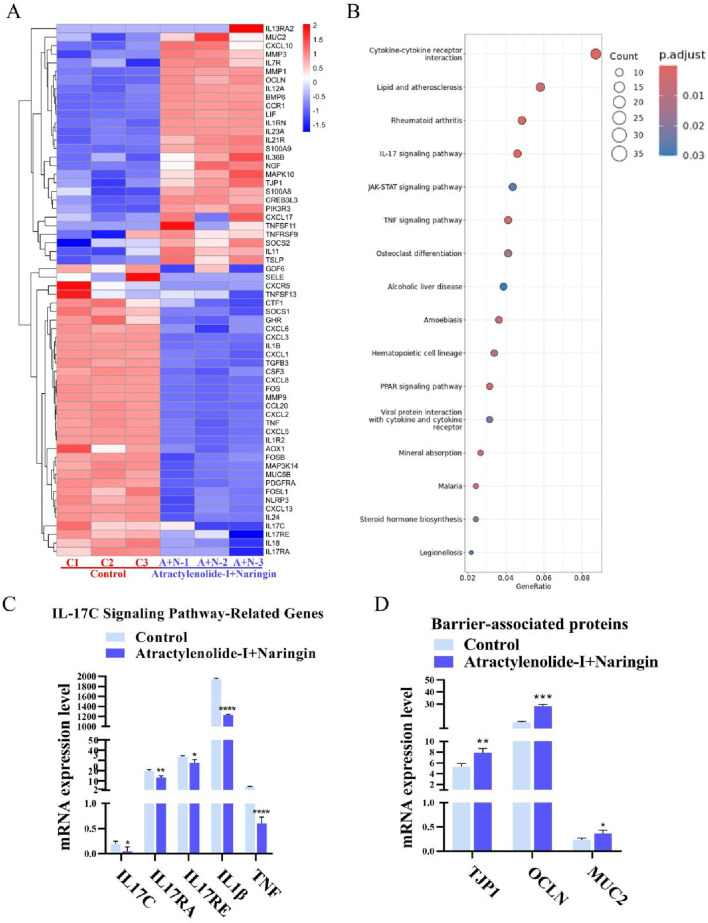
Gene expression of intestinal inflammation model after treatment. **(A)** Sample-specific mRNA expression heatmap. **(B)** KEGG enrichment analysis of mRNA-seq data. **(C)** mRNA expression levels of IL-17C signaling pathway-related genes (n = 3). **(D)** mRNA expression levels of barrier-associated proteins (n = 3).

Based on the intestinal protective effects and safety profile common to all the tested TCM and extracts, we performed mRNA sequencing (mRNA-seq) analysis on the most potent combination—the control and combined treatment groups (Atractylenolide-I + Naringin). As shown in Figure 6A, the combined treatment group exhibited downregulation of multiple pro-inflammatory factors, including TNF, IL1β, IL18, CXCL8, and CXCL3. Additionally, KEGG enrichment analysis identified several relevant pathways (Figure 6B), among which the IL-17C signaling pathway has been shown in previous studies to be closely associated with (IBD) (Van den Bossche et al., 2016). Key genes within the IL-17C pathway demonstrated significant downregulation in the treatment group (Figure 6C). Moreover, mRNA levels of multiple barrier-associated proteins were markedly upregulated in the treatment group (Figure 6D).

### 3.8 IL-17C-mediated positive feedback loop in the intestinal-on-a-chip model

**FIGURE 7:**
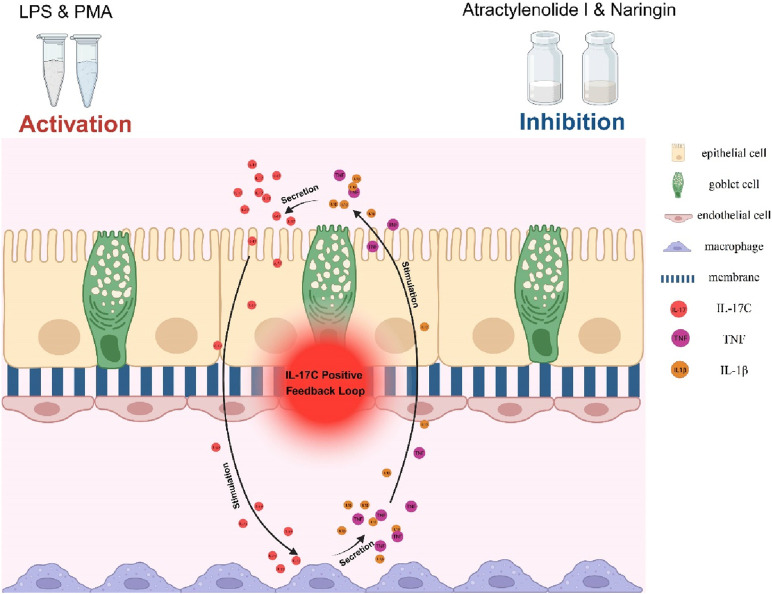
1L-17C positive feedback loop in intestinal chips. The self-reinforcing loop of “IL-17C → pro-inflammatory cytokines → IL-17C” drives the escalation of inflammatory reactions.

mRNA-seq analysis suggests the presence of an IL-17C-driven self-amplifying signaling circuit in the Intestinal-on-a-chip inflammatory model, which exacerbates inflammatory cascades through a cascade amplification mechanism. Upon co-stimulation with LPS and PMA, intestinal epithelial cells (IECs) and goblet cells were activated to secrete IL-17C (Li et al., 2000; Pappu et al., 2011; Iwakura et al., 2011). This cytokine subsequently bound to macrophage surface receptors, triggering the release of pro-inflammatory mediators such as TNF-α and IL-1β. Notably, these mediators further induced IL-17C secretion from epithelial cells, perpetuating the inflammatory cascade. We hypothesize that atractylenolide I and naringin may exert anti-inflammatory effects by targeting the IL-17C-mediated positive feedback loop (Figure 7).

## 4 Discussion

This study is the first to introduce an intestinal and liver organ-on-a-chip platform to systematically evaluate the effects of the TCM formula SFALCA and its active components atractylenolide I and naringin in treating intestinal inflammation. Following confirmation of its safety profile, further mechanistic investigations were conducted. We innovatively found that the combination of atractylenolide I and naringin provided better anti-inflammatory effects compared to each component alone, demonstrating significant therapeutic effects in an inflammatory bowel disease (IBD) model. Moreover, this study identified notable involvement of the IL-17 pathway in the TCM-based treatment of IBD.

First, we established both intestinal-on-a-chip and liver-on-a-chip models, and validated their functionality using multiple indicators to confirm their suitability for subsequent studies. In the intestinal model, a total of four cell types were used. The Caco-2 cell monolayer model is commonly used to simulate intestinal epithelium, but it typically differentiates into absorptive epithelium without mucus granules, failing to fully simulate the function of the intestinal mucus layer, which is crucial in drug absorption and permeability studies (Luis and Hansson, 2023; Johansson and Hansson, 2016). To overcome this limitation, we introduced HT-29-MTX cells into the intestinal organ-on-a-chip model, as these cells secrete mucus, further simulating the intestinal barrier function. Additionally, we co-cultured HUVEC endothelial cells and THP-1-derived macrophages. In this model, HUVEC cells significantly increased aminopeptidase catalytic activity, villin expression, and glycocalyx coverage, enhancing the physical barrier (Jing et al., 2020) THP-1-derived macrophages expressed corresponding cytokines after stimulation and treatment, simulating the development of inflammatory responses and the therapeutic effects.

Following model establishment, we evaluated the functionality through TEER measurements and relevant staining. Studies have shown that TEER values ≥ 250 Ω·cm^2^ indicate well-formed barrier integrity (Beduneau et al., 2014). In our developed Intestinal-immune chip, after the functional proteins were expressed in intestinal and hepatic cells, the TEER values reached over 400 Ω·cm^2^ (Figure 2B). Moreover, upon inflammatory stimulation with LPS and PMA, a rapid decrease in TEER was observed, confirming the model’s functional sensitivity. Immunofluorescence staining of ZO-1 and Alcian blue staining further verified the formation of intercellular tight junctions and the presence of a mucus layer (Figures 2C,D). In addition, normal expression of the HUVEC-specific marker CD-31 was detected (Figure 2D), and THP-1 macrophages exhibited characteristic M1/M2 polarization in subsequent experiments, supporting the potential of this Intestinal model for inflammation-related studies (Figure 2E).

Based on the optimal therapeutic concentration of 200 μg/mL (Supplementary Figure S2) determined through preliminary experiments, additional experimental groups incorporating multiple active components of TCM were included. In the anti-inflammatory study, we found that the raw atractylodes group did not exhibit significant anti-inflammatory effects, while the SFALCA group and its active components, atractylenolide I and naringin, whether used alone or in combination, exhibited significant anti-inflammatory effects. Specifically, ZO-1 expression increased, the proportion of M1 macrophages and pro-inflammatory cytokines were significantly reduced (Figures 4A–E). We hypothesize that the lack of significant anti-inflammatory effects in the raw atractylodes group may be due to its lower content of active ingredients. Literature indicates that atractylenolide I has anti-inflammatory effects in the concentration range of 25–100 μg/mL. We hypothesize that the lack of significant anti-inflammatory effects in the raw atractylodes group may be due to the low content of its active component, atractylenolide I. According to studies, the content of atractylenolide I in raw atractylodes is approximately 0.014%, whereas after processing, the content of atractylenolide I in SFALCA can increase by 1.2–1.7 times compared to raw atractylodes. Literature reports that the volatile oil content of atractylodes decreases after processing, while the lactone content increases significantly, and the synergistic effect of these two anti-inflammatory components enhances its anti-inflammatory effects. We also innovatively found that the combination of atractylenolide I and naringin provided better anti-inflammatory effects than when either component was used alone. Additionally, we observed no significant increase in the proportion of M2 macrophages in any drug-treated group, which we hypothesize may be due to the activation of M1 macrophages inhibiting their polarization to the anti-inflammatory M2 macrophages, possibly through mitochondrial dysfunction blocking the M1 to M2 polarization process (Van den Bossche et al., 2016).

Building upon the validated functionality of the TCM formulation and its active components, a liver organ-on-a-chip model was subsequently established by co-culturing HepG2 cells, LX-2 cells, and liver sinusoidal endothelial cells (LSECs) to systematically evaluate the hepatotoxicity of SFALCA and its extracts. Studies show that liver organ-on-a-chip systems are more accurate in detecting hepatotoxicity compared to traditional animal models and other *in vitro* models (such as 2D cells and 3D cell spheroids) (Ewart et al., 2022), especially in the context of multi-cell dynamic co-culture, providing results that are closer to the real physiological environment. Similarly, the fundamental functionality of the liver-on-a-chip model was validated by confirming the expression of key protein markers, including CD31, albumin, and α-SMA (Figure 5A). In the drug treatment evaluation, the known anti-inflammatory agent ALA and the hepatotoxic drug tolcapone were employed as positive controls. Results indicated that ALA effectively alleviated inflammation (Figures 4A–E), whereas tolcapone significantly reduced both the expression and activity of albumin (Figure 5C), one of the most sensitive markers for hepatotoxicity detection, thus accurately reflecting its hepatotoxic effect in the model. This highlights the specificity and accuracy of the platform in drug efficacy assessment. The study demonstrated that neither the TCM formulation nor its extracts induced a decrease in cell viability or albumin secretion in the liver model, indicating a favorable safety profile. Interestingly, SFALCA was found to exhibit notable hepatoprotective effects. This protective role, also observed with naringin which similarly increased albumin secretion, along with existing literature reports (Zhu et al., 2024), suggests that the hepatoprotective effect may originate from naringin. Further investigation is warranted to elucidate the specific underlying mechanisms.

Based on the evaluated anti-inflammatory efficacy in the intestinal model and demonstrated hepatic safety profile of the TCM formulation and its extracts, we further investigated the underlying anti-inflammatory mechanisms. Through KEGG pathway enrichment analysis of mRNA sequencing data from the combination treatment group using the two most effective TCM extracts, we identified significant involvement of the IL-17 signaling pathway in IBD treatment (Figure 6B). Genes associated with the IL-17 signaling pathway were markedly enriched in the sequencing results, with the treatment group showing significantly reduced expression levels of IL-17C, IL-17RA, IL-17RE, IL-1β, and TNF-α (Li et al., 2000). Multiple studies have demonstrated that IL-17C mRNA levels are markedly elevated in inflamed intestinal tissues. Both intestinal epithelial cells and goblet cells secrete IL-17C (Friedrich et al., 2015), which acts via paracrine signaling on the IL-17RA/IL-17RE heterodimer receptors of adjacent immune cells, further activating these cells and promoting macrophage secretion of TNF-α and IL-1β (Johansen et al., 2011; McGeachy et al., 2019; Song et al., 2011). These pro-inflammatory cytokines reciprocally stimulate intestinal epithelial cells, re-inducing IL-17C expression and forming a “IL-17C → pro-inflammatory cytokines → IL-17C” positive feedback loop, leading to excessive release of inflammatory mediators. Based on mRNA sequencing results, compared to the untreated group, the atractylenolide I and naringin combination therapy significantly reduced IL-17C, IL-17RA, IL-17RE, IL-1β, and TNF-α expression in the treatment group (Song et al., 2011). Concurrently, the restoration of tight junction proteins (ZO-1, occludin) (Figure 6D) suggests that the therapeutic agents inhibit IL-17C synthesis and secretion, thereby disrupting the inflammatory positive feedback loop, alleviating intestinal inflammation, and restoring epithelial barrier function (Chang et al., 2011).

This study has certain limitations. The current model uses cell lines rather than primary organoids, limiting its physiological relevance. Additionally, while mRNA seq suggests IL-17C involvement, protein-level validation is still required. Further study of naringin should include more biochemical and histological data. Future efforts will focus on developing a multi-organ microphysiological system integrating intestinal, hepatic, pulmonary, and cardiac models to better simulate human physiology. Such a platform will provide a more comprehensive approach for evaluating the multi-target mechanisms of TCM.

## 5 Conclusion

This study established an intestinal and liver organ-on-a-chip platform to evaluate the effects of SFALCA on intestinal inflammation treatment and liver toxicity. The results show that SFALCA exhibits significant anti-inflammatory effects without liver toxicity, with the main active ingredients being atractylenolide I and naringin. Moreover, it was innovatively found that the combined effect of naringin and atractylenolide I is superior to using each component alone. In addition to the preliminary elucidation of the anti-inflammatory mechanisms of SFALCA, this study also provides a novel technical platform and research methodology for investigating the toxicity and pharmacological mechanisms of TCM.

## Data Availability

The data presented in the study are deposited in the NGDC - GSA for Human Genome Sequence Archive, accession number HRA016183. Available at: https://ngdc.cncb.ac.cn/gsa-human/browse/HRA016183.
