# Supplementary material for: Mechanisms and safety evaluation of stir-fried atractylodis macrocephalae rhizoma with aurantii fructus in the treatment of inflammatory bowel disease: a study based on organ-on-a-chip model
Source: Front Pharmacol. 2026 Jan 29;16:1708719. doi: 10.3389/fphar.2025.1708719 (PMC12894399; doi:10.3389/fphar.2025.1708719)
Supplement: Supplementary file 1 [file DataSheet1.docx]

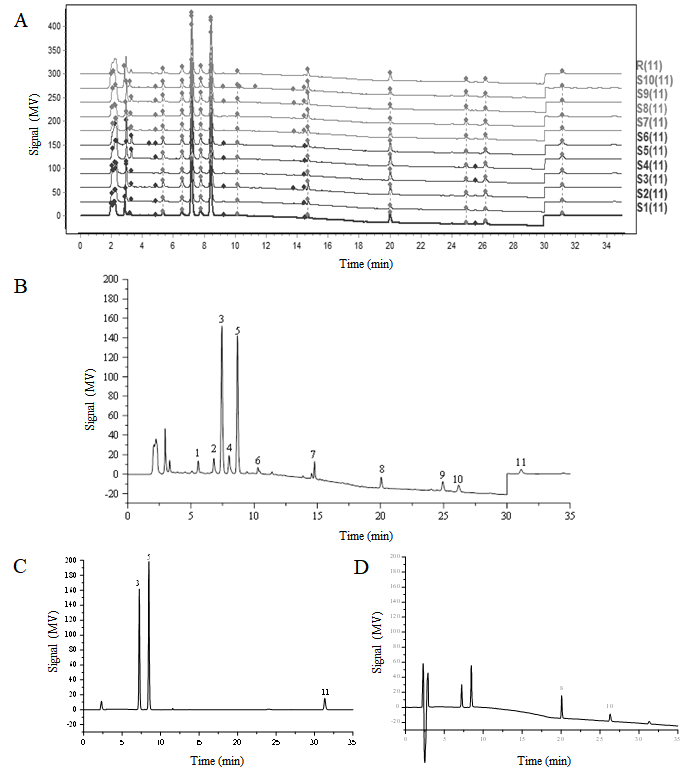


**Figure S1. Representative fingerprints** **of main ingredients in SFALCA.** (A-B) SFALCA sample. (C-D) Mixing standards sample. Naringin (Peak 3), Neoeriocitrin (Peak 5)，Atractylenolide I（Peak 11）.


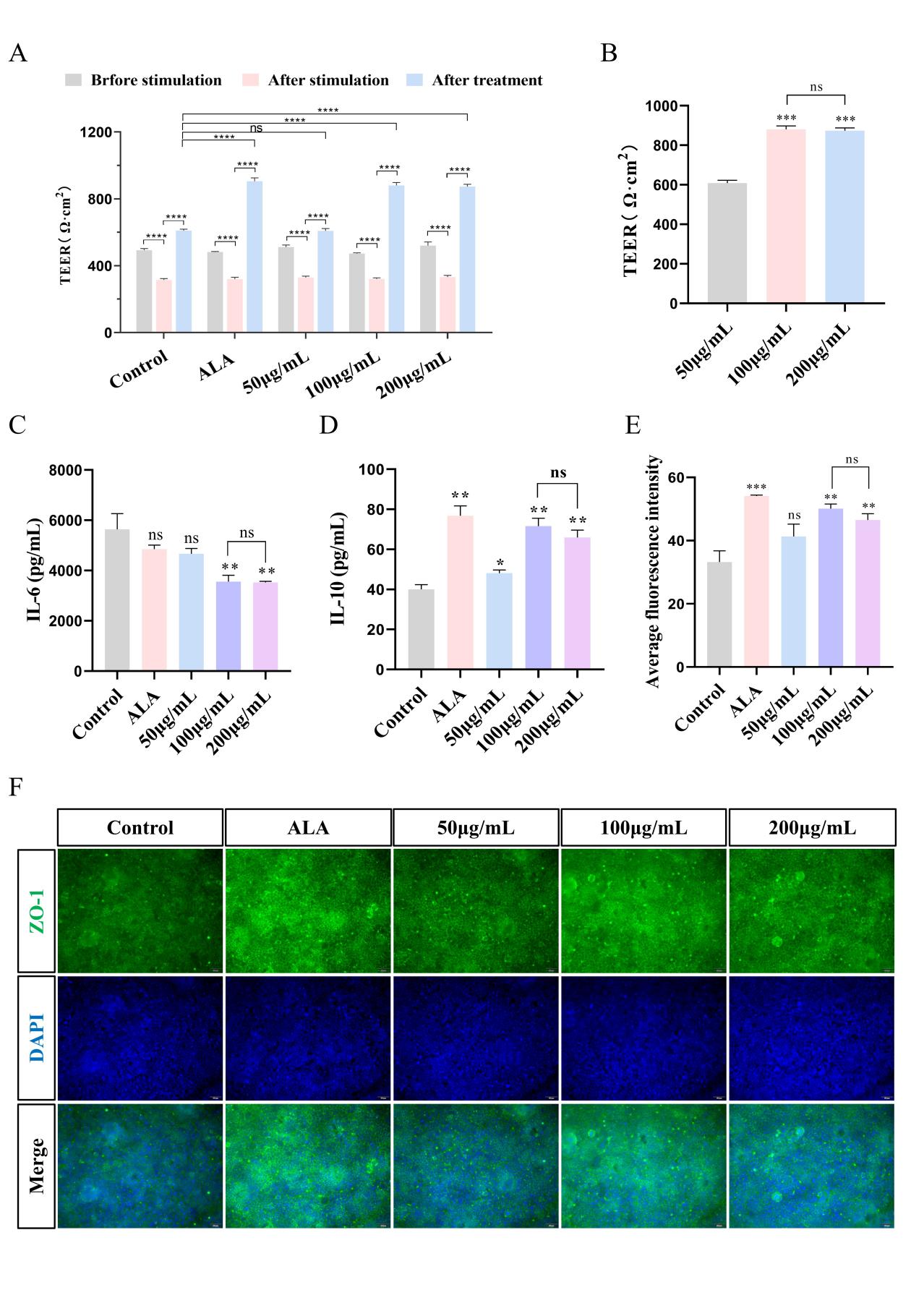


**Figure S2. Screening for the optimal concentration of SFALCA.** (A-B) TEER values in each group. (C-D) ELISA kits were used to analyze the concentrations of IL-6 and IL-10 in each group (n = 3). (E-F) Immunofluorescence staining results of ZO-1 and statistical analysis in each group (n = 3). **p* < 0.05; ***p* < 0.01; ns, not significant. Data are presented as mean±SD.
